# How does mandibular advancement with or without maxillary procedures affect pharyngeal airways? An overview of systematic reviews

**DOI:** 10.1371/journal.pone.0181146

**Published:** 2017-07-27

**Authors:** Su Keng Tan, Wai Keung Leung, Alexander Tin Hong Tang, Roger A. Zwahlen

**Affiliations:** 1 Center of Oral & Maxillofacial Surgery Studies, Faculty of Dentistry, Universiti Teknologi MARA Sungai Buloh Campus, Jalan Hospital, Selangor Darul Ehsan, Malaysia; 2 Discipline of Oral and Maxillofacial Surgery, Faculty of Dentistry, University of Hong Kong, Hong Kong SAR, PR China; 3 Discipline of Periodontology, Faculty of Dentistry, The University of Hong Kong, Prince Philip Dental Hospital, Hong Kong SAR, PR China; 4 Orthodontist in private practice, Central, Hong Kong SAR, PR China; UNITED STATES

## Abstract

**Background:**

Mandibular advancement surgery may positively affect pharyngeal airways and therefore potentially beneficial to obstructive sleep apnea (OSA).

**Objective:**

To collect evidence from published systematic reviews that have evaluated pharyngeal airway changes related to mandibular advancement with or without maxillary procedures.

**Methodology:**

PubMed, EMBASE, Web of Science, and Cochrane Library were searched without limiting language or timeline. Eligible systematic reviews evaluating changes in pharyngeal airway dimensions and respiratory parameters after mandibular advancement with or without maxillary surgery were identified and included.

**Results:**

This overview has included eleven systematic reviews. Maxillomandibular advancement (MMA) increases linear, cross-sectional plane and volumetric measurements of pharyngeal airways significantly (p<0.0001), while reducing the apnea-hypopnea index (AHI) and the respiratory disturbance index (RDI) significantly (p<0.0001). Two systematic reviews included primary studies that have evaluated single-jaw mandibular advancement, but did not discuss their effect onto pharyngeal airways. Based on the included primary studies of those systematic reviews, single-jaw mandibular advancement was reported to significantly increase pharyngeal airway dimensions (p<0.05); however, conclusive long-term results were lacking.

**Conclusion:**

MMA increases pharyngeal airway dimensions and is beneficial to patients suffering from OSA. However, more evidence is still needed to draw definite conclusion related to the effect of single-jaw mandibular advancement osteotomies on pharyngeal airways.

## Introduction

Pharyngeal airway dimensions are inevitably affected by skeletal jaw movements during orthognathic surgery. Both one-jaw mandibular advancement[1, 2] and two-jaw maxillomandibular advancement (MMA)[2] have been reported to increase pharyngeal airway dimensions. The one-jaw approach is less popular because two-jaw osteotomy provides an overall more balanced post-surgical aesthetic outcome. Furthermore, aside from being used to treat certain dentofacial deformities, two-jaw osteotomies have also been reported to be effective in treating or reducing the severity of obstructive sleep apnea (OSA)[3].

Surgeons and orthodontists have gained increasing interest in pharyngeal airway evaluation, as it affects patients’ health and quality of life[3]. The effects of orthognathic procedures on pharyngeal airways were commonly assessed by analyzing cephalometric images[1, 4, 5]. Recently, 3-dimensional (3-D) imaging, i.e. cone-beam computed tomography (CBCT)[2, 6], computed tomography (CT)[7] and magnetic resonance imaging (MRI)[8] have become more and more important within this research field. To date, some systematic reviews[3, 9–18] reported on pharyngeal airway anatomical and/or respiratory parameter changes after mandibular advancement surgery and supported the benefit of mandibular advancement on OSA. However, the definite anatomical and physiological changes in pharyngeal airways after mandibular advancement are still not established. Therefore, an overview of systematic reviews in this topic is important to analyze and summarize the reported data, and to identify any weakness, inconsistency or research gaps in this particular field.

The aims of this overview were to examine systematic reviews for changes in pharyngeal airway dimensions and/or respiratory parameters related to mandibular advancement osteotomies with or without concomitant maxillary osteotomies, and to critically appraise the quality of the reported systematic reviews.

## Methodology

The reporting of this systematic review adheres to the Cochrane’s recommendation on overview of systematic reviews[19] and the Preferred Reporting Items for Systematic Reviews and Meta-analyses (PRISMA) statement[20, 21] when relevant. A review protocol was developed and registered with PROSPERO; registration number: CRD42016046489 (https://www.crd.york.ac.uk/prospero/display_record.asp?ID=CRD42016046489).

### Search method

The electronic databases PubMed, EMBASE, Web of Science, Scopus and Cochrane Library were searched using the search strategy outlined in Table 1. The Web of Science database search has included the search of both journals and proceedings. The last search was performed on 23^rd^ April 2017. There was no search limitation set for publication language or dates. The search results were exported into Endnote X7 (Thomson Reuters, CA, USA) and duplicate articles were removed. Next, the title and abstract of all articles were screened for potential eligibility, and the full text of relevant articles was retrieved. Lastly, the reference lists of those relevant articles were manually searched to screen for further relevant articles. Both electronic and manual searches were performed independently by two authors (TSK and RAZ). Disagreement was resolved by discussion with the other authors.

**Table 1:** Electronic databases search strategy (refer to S1 Text for detailed search strategy).

Electronic databases	Search strategy
PubMed	(Systematic review OR review OR overview OR meta-analysis OR evidence based medicine OR evidence based dentistry OR review literature OR literature review)
EMBASE	AND
Web of Science	(orthognathic surgery OR orthognathic surgical procedure OR orthodontics surgery OR maxillomandibular advancement OR mandibular surgery OR maxillary surgery OR bimaxillary surgery OR jaw surgery OR surgical orthodontic treatment OR jaw advancement OR jaw movement OR mandibular advancement)
Cochrane library	AND
Scopus	(upper airway OR pharynx OR pharyngeal OR oropharynx OR oropharyngeal OR nasopharynx OR nasopharyngeal OR hypopharynx OR hypopharyngeal)

### Selection of reviews

This overview has included systematic reviews that have assessed changes of pharyngeal airways related to mandibular advancement osteotomies with or without concomitant maxillary osteotomies. Eligible systematic reviews had to report outcome measures of pharyngeal airway dimensions and their post-surgical changes, i.e. linear, cross-sectional plane or volumetric measurements. Furthermore, data from reviews reporting on respiratory parameter changes have also been evaluated and included.

Systematic reviews that have studied specific target group (i.e. edentulous patients and morbidly obese OSA patients), or pharyngeal airways in cleft lip and palate, syndromic or distraction osteogenesis patients have been excluded from this overview.

### Data extraction and management

Data from included systematic reviews was extracted independently by two authors (TSK, RAZ) and inserted in pre-tabulated data sheets (Excel, Microsoft, New Mexico). Any disagreement related to data extraction was resolved by consensus in discussion with the other authors (LWK, TTH) to ensure consistency and reliability of extracted data. The data extraction included authors, publication year and title, methods of analyses, number and study design of included studies, sample population (number, age and gender of patients); type of interventions, outcome measures and main findings, follow up period and meta-analyses’ result when available.

### Assessment of methodological quality of included reviews

The methodological quality of the included reviews was assessed independently by TSK and RAZ using the Assessment of Multiple Systematic Reviews (AMSTAR) tool[22]. On the other hand, quality of evidence of primary studies included in the systematic reviews was appraised based on the particular assessment technique being used by each systematic review. Discussion among all authors was used to resolve any disagreement.

### Data synthesis and statistical analysis

A narrative overview is provided summarizing the data gathered from included systematic reviews. Meta-analyses have been performed whenever possible by pooling the data across different reviews using the software “Review Manager” (RevMan version 5.3; Copenhagen: Nordic Cochrane Center, Cochrane Collaboration; 2014). The heterogeneity of trial results was assessed with the χ^2^ test for heterogeneity (p = 0.1) and the *Ι*^*2*^ measure of inconsistency. A significant heterogeneity was considered when p< 0.1 for χ^2^ test or when *Ι*^*2*^> 50%. Treatment effects across the studies were combined using the fixed effect model when there was no heterogeneity observed (p> 0.1); in case of heterogeneity observed, the random effect model was applied. Funnel plot was used to assess publication bias, while Egger test for funnel plot asymmetry will be used when more than ten primary studies were included in an analysis[23, 24].

## Results

### Quantity of current evidence

**Fig 1:**
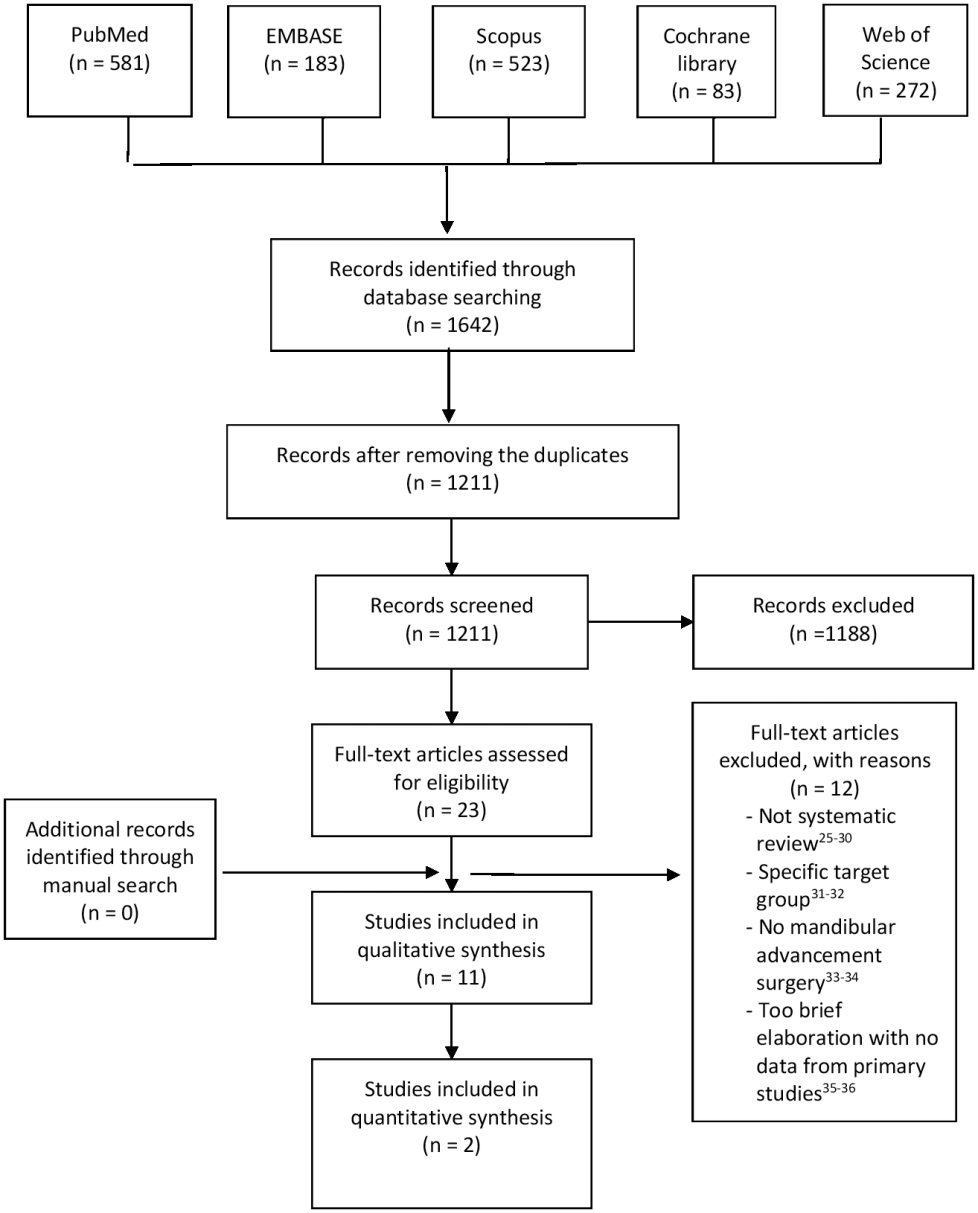
Study selection process.

An electronic search of the databases has generated an overall of 1642 articles. Titles and abstracts of 1211 articles were screened after removing the duplicates. The full texts of 23 relevant articles were retrieved and assessed for their eligibility of inclusion. No other relevant article was found while manually searching the reference lists of those 23 articles. Ultimately, 11 systematic reviews [3, 9–18] have been found to match both inclusion and exclusion criteria after eliminating 12 articles[25–36]. The study selection process is summarized in Fig 1.

Two systematic reviews[11, 16] have reported effects of various orthognathic surgical procedures onto pharyngeal airways, while eight others[9, 10, 12–15, 17, 18] have focused on MMA and other procedures within the scope of OSA treatment. There was only one review[3] reported about the effect of MMA in both OSA and non-OSA studies. The characteristics of the included articles are highlighted in Table 2. There were two systematic reviews[11, 16] focused on pharyngeal airway analyses, four reviews[10, 12, 15, 17] only analyzed changes in respiratory parameters, and the others[3, 9, 13, 14, 18] evaluated both outcomes.

**Table 2:** Characteristics of included articles.

Authors, year	Type of review	Database searched	Primary studies										
			Airway assessed	Included studies		Participants			Patients	Interventions (Number of studies)	Outcome measures		Maximum follow-up period *Range*
				Studies with mandibular advancement /Total number of primary studies	Type	Total number *(Range)*	M/F	Age (Range of mean) *years old*			Airway parameters (Measurement methods)	Respiratory parameters	
Alsufyani *et al*, 2013[9]	SR	Medline, EBM^#^, Scopus	OP; partial NP	4/7	1 CS; 3 CR	7 (1–4)	4/3	Mean: 43	SDB/ OSAS	MMA(4)	Total volume, MCS area, linear, total pressure drop, airway resistance (CBCT)	AHI, Sleep Q Epworth sleepiness scale	7 weeks-6 months *(NR*: *1 study)*
Caples *et al*, 2010[10]	MA	(MEDLINE, EMBASE, Current Contents, Cochrane CENTRAL through Ovid)	NR	9/70	9 O	234 *(NR)*	9:1 *(ratio)*	43.9 (41–48)	OSA	MMA (9)	NR	AHI	NR
Christovam *et al*, 2016[11]	MA	Cochrane library, Medline, Scopus, VHL, Web of Science, Open-Grey.	UA	11/27	9 R; 2 P	252 (9–102)	102/112 *(NR*:*2 studies)*	22–44.6	NR	MMA (9); MdA (1); MMA/MdA (1)	Cross-sectional area and volume (10 CBCT/CT; 1 MRI)	NR	5 weeks-1 year *(NR*: *1 study)*
Elshaug*et al*, 2007[12]	MA	Medline	NR	4/18	4 CS	38 (5–15)	NR	NR	OSA	MMA (3) MMA/MdA (1)	NR	AHI	NR
Hsieh and Liao, 2013[14]	SR	PubMed	PAS	15/15	15 CS (2P, 13R)	376 (11–50)	219/ 157	33–51	OSA	MMA (15)	Linear (posterior airway space, airway length); Vertical hyoid position (CT and ceph)	AHI, RDI, LSAT	6 weeks– 21.8 months
Holty and Guilleminault, 2010[13]	MA	Medline	PAS	59/59	CS; CR	957 (1–175)	836/ 121	Mean: 44.4±9.4	OSA	MMA (59)	PAS (Ceph)	AHI, SpO_2_ nadir	3–7.7 months
Knudsen *et al*, 2015[15]	MA	PubMed, Cochrane	PAS	4/4	NR	115 (17–40)	NR	NR	OSA	MMA (4)	NR	AHI, LSAT	NR
Mattos *et al*, 2011[16]	MA	VHL, Scirus, Ovid, SIGLE.	OP	5 ^β^/22	4 R 1P	135 (12–58)	40/83 *(NR*:*1 study)*	21–36.3 *(NR*:*1 study)*	NR	MdA(3); MMA(2)	Linear (Ceph)	NR	6 weeks– 12 years
Rosario *et al*, 2016[3]	MA	PubMed, Scopus, Science Direct, SciELO	UA	7 ^α^/7	CCT	103 (10–25)	47/46 *(NR*:*1 study)*	22–42.75 *(NR*:*1 study)*	OSA (4); Others (3)	MMA (7)	Volume (CT)	AHI	2–49 months *(NR*: *1 study)*
Pirklbauer *et al*, 2011[17]	SR	PubMed	NR	28/28	1 RCT 5 Co 22 CS/CR	917 (3–175)	561/87 (*NR*: *269 patients)*	41.0–50.3	OSA	MMA (28)	NR	AHI, RDI	2–12 months *(NR*: *1 study)*
Zaghi *et al*, 2016[18]	^δ^MA	Cochrane library, Scopus, Web of Science, Medline	PAS	45/45	1 RCT *(NR*: *44 studies)*	518 (1–35)	282/57 *(NR*: *179 patients)*	Mean: 45.3 ±10	OSA	MMA (45)	Linear (Ceph)	AHI, RDI	2–6 months

^#^ EBM = All evidence-based medicine reviews (EBM), including Cochrane Database

^β^ 2 articles[5, 37] have included both mandibular advancement and setback surgeries.

^α^ Only 6 studies included in the meta-analysis (One study did not report absolute value for average on difference)

^δ^ Meta-analysis was only performed on respiratory parameters only

**Abbreviations:** SR = systematic review; MA = meta-analysis; NP = nasopharyngeal, OP = oropharyngeal, HP = hypopharyngeal, UA = upper airways, PAS = Posterior airway space; R = Retrospective study; O = Observational, P = Prospective study; CCT = Case controlled trials; Co = Cohort study, CS = Case series; CR = Case report; SDB = Sleep disordered breathing; OSAS = obstructive sleep apnea syndrome; MdA = Mandibular advancement, MMA = Maxillomandibular advancement; MCS area = Minimum cross-sectional area; Ceph = Cephalometric; CT = Computed tomography; CBCT = Cone-beam computed tomography; MRI = magnetic resonance imaging; AHI = Apnea-hypopnea index; RDI = respiratory disturbance index; SpO_2_nadir = lowest oxyhemoglobin saturation measured during sleep; LSAT = lowest oxygen saturation, NR = not reported

### Quality of systematic reviews (AMSTAR)

The AMSTAR tool analysis revealed one systematic review[16]with a high score missing out only one item (Table 3). In general, systematic reviews[11, 16] assessing pharyngeal airways have the highest scores (mean = 9 “yes”), followed by reviews[3, 9, 13, 14, 18] assessed both pharyngeal airways and respiratory parameters (mean = 4.8 “yes). The systematic reviews[10, 12, 15, 17]analyzing only respiratory parameters have the lowest score (mean = 3.25 “yes”).

**Table 3:** Quality assessment of included systematic reviews with AMSTAR tool.

AMSTAR criteria	Alsufyani *et al*[9]	*Caples *et al*[10]	*Christovam *et al*[11]	*Elshaug *et al*[12]	Hsieh and Liao[14]	*Holty and Guilleminaul [13]	*Knudsen *et al*[15]	*Mattos *et al*[16]	*Rosario *et al*[3]	Pirklbauer *et al[*17*]*	*Zaghi *et al*[18]
1. Was an ‘a priori’ design provided?	CA	CA	Y	CA	CA	CA	CA	Y	Y	CA	CA
2. Was there duplicate study selection and data extraction?	Y	Y	Y	CA	Y	CA	CA	Y	Y	CA	Y
3. Was a comprehensive literature search performed?	Y	Y	Y	N	N	N	Y	Y	Y	N	Y
4. Was the status of publication (i.e. grey literature) used as an inclusion criterion?	N	N	Y	N	N	N	N	Y	N	N	N
5. Was a list of studies (included and excluded) provided?	N	N	N	N	N	Y^β^	N	Y	Y	N	N
6. Were the characteristics of the included studies provided?	Y	Y	Y	N	N	Y	N	Y	Y	Y	Y
7. Was the scientific quality of the included studies assessed and documented?	Y	N	Y	Y	Y	N	N	Y	Y	Y	Y
8. Was the scientific quality of the included studies used appropriately in formulating conclusions?	N	NA	Y	Y	Y	NA	NA	Y	N	Y	N
9. Were the methods used to combine the findings of studies appropriate?	N	Y	Y	Y	Y	Y	Y	Y	Y	Y	Y
10. Was the likelihood of publication bias assessed?	N	N	N	N	N	N	N	Y	N	NA	Y
11. Was the conflict of interest stated?	CA	CA	CA	CA	CA	CA	CA	CA	CA	CA	CA
**TOTAL “YES”**	4	4	8	3	4	3	2	10	7	4	6

*Systematic reviews with meta-analysis;

^β^ Yes, but not complete list of all excluded studies

**Abbreviations:** Y = yes; N = no; CA = can’t answer; NA = not applicable

Although a self-declared no conflict of interest was found in eight systematic reviews[3, 9–13, 16, 18], none of them reported about the issue of conflict of interest of their included primary studies. Besides, only three reviews[3, 11, 16] reported a ‘priori’ design. While seven reviews[3, 9–11, 15, 16, 18] performed a comprehensive search of several databases, four[12–14, 17] screened only one database. On the other hand, three reviews[10, 13, 15] did not assess the quality of their included primary studies. While eight out of eleven systematic reviews have performed meta-analyses, only two[16, 18] of them declared on their assessment of publication bias.

### Quality of evidence from primary studies in included reviews

Eight out of eleven systematic reviews have analyzed the quality of evidence of their included primary studies. Three reviews[3, 9, 11] assessed the risk of bias; three others[14, 16, 18] evaluated the quality of methodology of their primary studies; and two reviews[12, 17] reported on the level of evidence (Table 4). Although the quality assessment of primary studies is an essential methodological step in systematic reviews, two reviews[13, 15] did not mention it, while another one[10] only performed group analysis based on the type of procedures. Not all primary studies have been analyzed quantitatively in the eight meta-analyses. Hence, only the primary studies involved in quantitative analyses in these papers have been evaluated in this section.

**Table 4:** Quality assessment for primary studies of included systematic reviews.

Systematic reviews	Assessment method	Assessment criteria	Scoring method	Result	Remark
Alsufyani *et al*, 2013[9]	Risk of bias assessment with customized tool adopted from *AHRQ EPC Methods Guide[38]	Selection bias Detection or measurement bias Analysis or interpretation bias Performance bias	High risk of bias (<50%) Moderate risk of bias (50%) Low risk of bias (>50%)	7 High risk of bias	Pilot testing of the tool was performed. Result mostly due to criteria 1 and 2.
Christovam *et al*, 2016[11]	Risk of bias based on quality assessment method reported by Mattos *et al*[16]	Eligible criteria for participants described Presence of control group Blinding assessment stated Statistical treatment performed Reliability of measures tested Reporting drop-outs Follow-up period reported Potential bias and trial limitations addressed	Low risk of bias (≥4.5) Moderate risk of bias (>2 and <4.5) High risk of bias (≤2)	1 Low risk of bias 6 Moderate risk of bias	High risk papers have been excluded from the review
Elshaug *et al*, 2007[12]	Level of evidence	Type of publication	Level 1: systematic review of or individual randomized, controlled trial or trials (RCT) Level 2: cohort study Level 3: case-control study Level 4: case series Level 5: expert opinion	4 Level 4	-
Hsieh and Liao, 2013[14]	Methodology soundness checklist (modified from Antczak *et al*[39] and Jadad *et al*[40])	Study design Sample size Method of selection Consecutive recruitment Valid methods Consideration of confounding factors Analysis of errors in methods ‘blinding’ in measurement Adequate statistical analysis	Low quality (≤3) Medium quality (4–7) High quality (8–9)	10 Low quality 5 Medium quality	-
Mattos *et al*, 2011[16]	Self-compiled criteria for quality of methodological soundness (mostly based on CONSORT statement)	As above (refer Christovam *et al*)	High quality (>6 points) Moderate quality (4–6 points) Low quality (<4 points)	2 Moderate quality	Low research quality of methodological soundness studies were excluded from the review.
Priklbauer *et al*, 2011[17]	Criteria defined by the Oxford Centre of evidence-based medicine	1a Systematic review of randomized controlled trials 1b Individual randomized controlled trial 2a Systematic review of cohort studies 2b Individual cohort study 3a Systematic review of case control studies 3b Individual case control studies 4 Case series/case report 5 Expert opinion, bench research	Grade A: level 1a, 1b Grade B: level 2a, 2b, 3a, 3b Grade C: Level 4 Grade D: Level 5	1 Grade A 5 Grade B 22 Grade C	-
Rosario *et al*, 2016[3]	Risk of bias across studies (checklist adapted from Cericato *et al*[41])	Clear abstract Clear and precise objective Cited ethical aspects Adequate research design Reported sample size calculation Control group presence Cited statistical methods Clear and precise results Study limitation discussed	Low quality (0–6 points) Medium quality (7–9 points) High quality (10–12 points)	1 High quality 6 Moderate quality	3 low quality articles were excluded
Zaghi *et al*, 2016[18]	Methodology quality assessment questionnaire (self-developed)	Clinical description and characteristics (4 items) Sleep study test quality Independence of sleep study interpretation Surgical technique quality Sample size Cohort assembly	1 point for each “yes” 0 point for each “no” Scores 0–10	Mean: 5.11±1.43 Median: 5 Range: 2–8	Larger sample size was not significantly associated with higher quality (p = 0.5102)

* AHRQ EPC Methods Guide = Agency for Healthcare Research and Quality (AHRQ) Evidence-based Practice Center (EPC) Methods Guide for Effectiveness and Comparative Effectiveness Reviews on assessing the risk of bias of individual studies.

- Quality of primary studies was not assessed or incomplete in three systematic reviews[10, 13, 15]

For the 38 primary studies that have been assessed based on risk of bias or methodology quality, only two were reported as low risk of bias or high methodological quality. Besides, half of them showed a moderate risk of bias or methodological quality. Zaghi *et al*[18] did not rate the quality of their primary papers, but have instead calculated the mean quality scores (5.11±1.43; range: 2–8).

### Outcome measures

The narrative information from the included systematic reviews is elaborated below. The results of the meta-analyses of included systematic reviews are shown in Table 5.

**Table 5:** Results from multiple meta-analyses of MMA procedures reported by included systematic reviews.

Meta-analyses	Outcome measure(s)	Results	No of studies (No. of patients)
***Airway parameters*:**			
Christovam *et al*, 2016**[**11**]**	mCSA changes	Increased significantly *(p = 0.000), MD = 124.13mm^2^; I^2^ = 43%	3 (29)
		*(Two studies were removed from the analysis to reduced* I^2^ *from 84% to 43%)*	
		#Remark: Also found significant increase at retropalatal (mean = 118.63mm^2^) and retrolingual (mean = 94.84mm^2^)	
	Total volume changes	Increased significantly *(p = 0.000), MD = 7416.10mm^3^; I^2^ = 0%	5 (66)
		*(Three studies were removed from the analysis to reduced* I^2^ *from 98% to 0%)*	
		#Remark: Also found significant increase at retropalatal (mean = 727.44mm^3^) and retrolingual (mean = 2530.05mm^3^)	
Mattos *et al*, 2011[16]	AP changes (soft palate-pharyngeal wall)	Increased significantly (p<0.00001), MD = 3.64mm [95% CI 2.67, 4.61]; I^2^ = 0%	2 (88)
Rosario *et al*, 2016[3]	UA volume changes	Increased significantly (p<0.00001), MD = 7.86ml [95% CI 5.47, 10.07]; I^2^ = 0%	6 (83)
***Respiratory parameters*:**			
Caples *et al*, 2010[10]	AHI reduction %	Ratio of means [mean = 0.13; 95% CI 0.08, 0.200]; p<0.00001; *I*^2^ = 91%	9 (234)
Elshaug *et al*, 2007[12]	Surgical success rate	1. 86% [95% CI 0.74, 0.95] for 50% AHI reduction/ AHI ≤ 20/h / both3.	4 (38)^γ^
		2. 45% [95% CI 0.30, 0.60] for AHI ≤ 10/h	
		3. 43% [95% CI 0.28, 0.58] for AHI ≤ 5/h	
Holty *et al*, 2010[13]	AHI changes	Reduced significantly (p<0.001); Mean = 63.9±26.7/h vs 9.5±10.7/h	22 (627)
	SpO2 nadir	Increased significantly (p<0.001); Mean = 71.9+14.8% versus 87.7+4.8%	17 (516)
	Surgical success rate	86% for 50% AHI reduction/ AHI ≤ 20 / both43.2% for AHI<5/h	22 (627)
		77.6% for AHI < 15/h	
		63.4% for AHI <10/h	
		43.2% for AHI<5/h	
Knudsen *et al*, 2015[15]	AHI changes	Mean OR = 14.9 [95% CI 2.7, 83.5]; p = 0.002; *I*^2^ = 0% for AHI ≤ 5	3 (49)
		Mean OR = 114.8 [95% CI 23.5, 561.1]; p<0.00001; *I*^2^ = 0% for AHI ≤ 20	4 (59)
		Mean OR = 6.09 [95% CI 2.18, 16.96]; p<0.00001; *I*^2^ = 48% for AHI decrease >50%	3 (36)
Zaghi *et al*, 2016[18]	AHI changes	Reduced significantly (p<0.001), MD = -47.8/h [95% CI ±4.7]; *I*^2^ = 61.3%	36 (455)
	RDI changes	Reduced significantly (p<0.001), MD = -44.4/h [95% CI ±8.0]; *I*^2^ = 41.3%	11 (68)

*The article only reported up to three decimal digits

^#^The article only described the result in text without figure, thus some data like p-value was missing.

^γ^ 4 cases were mandibular advancement only, other 34 cases were MMA.

**Abbreviations:** mCSA = minimum cross sectional area; MD = mean difference; UA = upper airway; AHI = apnea-hypopnea index; RDI = respiratory disturbance index; SpO2 nadir = lowest oxyhaemoglaobin saturation measured during sleep; OR = odd ratio

Although two systematic reviews[11, 16] included five primary studies[1, 2, 4–6] that have assessed the effect of isolated mandibular advancement on pharyngeal airways, no further data elaboration was performed. Therefore, full articles for these five primary studies were retrieved and their findings were briefly summarized in Table 6.

**Table 6:** Summary from primary studies of isolated mandibular advancement osteotomies^#^.

Primary studies	Paticipants (M:F)	Mean Age (Range) *years old*	Maximum follow-up period	Imaging method	Main findings
Archilleos *et al*., 2000[1]	20(20:-)	26.27(17.33–43.58)	PO 3 years	Ceph	PO 6 months: Significant larger PA (sagittal dimension) at the level of OP (p<0.05) and tongue base (p<0.01) PO 3 years: Significant wider minimum dimension of PA (P<0.05) Long term (3 years) widening of minimal PA space
Eggensperger *et al*.,2005[4]	15(4:11)	21(17–31)	PO 12 years	Ceph	Immediate PO: increased of UP and LP size PO 1 year: MP smaller than pre-op PO 12 years: both UPA and MPA significantly (p<0.05) smaller than pre-op; LPA returned to pre-op value Mandibular advancement surgery alone did not increase pharyngeal airway in long term (12 years)
Hernandez-Alfaro *et al*., 2011[2]	10	*	Mean:PO121.4 days	CBCT	Average PA space volume increase of 78.3% (range: 0.9–167.6%) Mandibular advancement will enlarge PA space volume
Kochel *et al*., 2013	102(27:75)	31.8	PO 5 weeks	CBCT	PO 5 weeks: Significant increased (p<0.001) at posterior NP (12.5%), upper OP (38.8%) and lower OP (45.6%) of cross-sectional area at the level of soft palate (48.5%), hard palate (14.6%), epiglottis tip (21.6%) and minimum cross-sectional area (46.9%) of diameters in both sagittal and transversal planes
Turnbull *et al*., 2000	8	*	PO 6 weeks	Ceph	*Unable to draw isolated finding for mandibular advancement procedures only as all results were analyzed based on mandibular ± maxillary advancement procedures in this review*.

^#^ All isolated mandibular advancement osteotomies here were bilateral sagittal split osteotomies (BSSO).

* Unable to be determined asthe study also involve other groups with different surgical procedures.

**Abbreviations:** M = male; F = female; Ceph = cephalometric; CBCT = con beam computed tomography; PA = pharyngeal airway; OP = oropharyngx; NP = nasopharynx; UPA = upper pharyngeal airway; MPA = middle pharyngeal airway; LPA = lower pharyngeal airway; pre-op = pre-operative; PO = post-operative.

#### Airway parameters

Meta-analyses of three systematic reviews showed a significant increase of minimum cross-sectional area (CSA)[11], pharyngeal airway volume[3, 11] and antero-posterior distance from the soft palate to the pharyngeal wall[16] after MMA (Table 5). Others[9, 18] reported an increased post-MMA pharyngeal airway volume as well as a minimum CSA when evaluating the data of their primary studies. Hsieh and Liao, 2013[14] revealed a significant increase of the posterior airway space at multiple measurement locations after MMA in all 14 primary studies, a finding that was supported elsewhere[13, 18].

**Fig 2:**
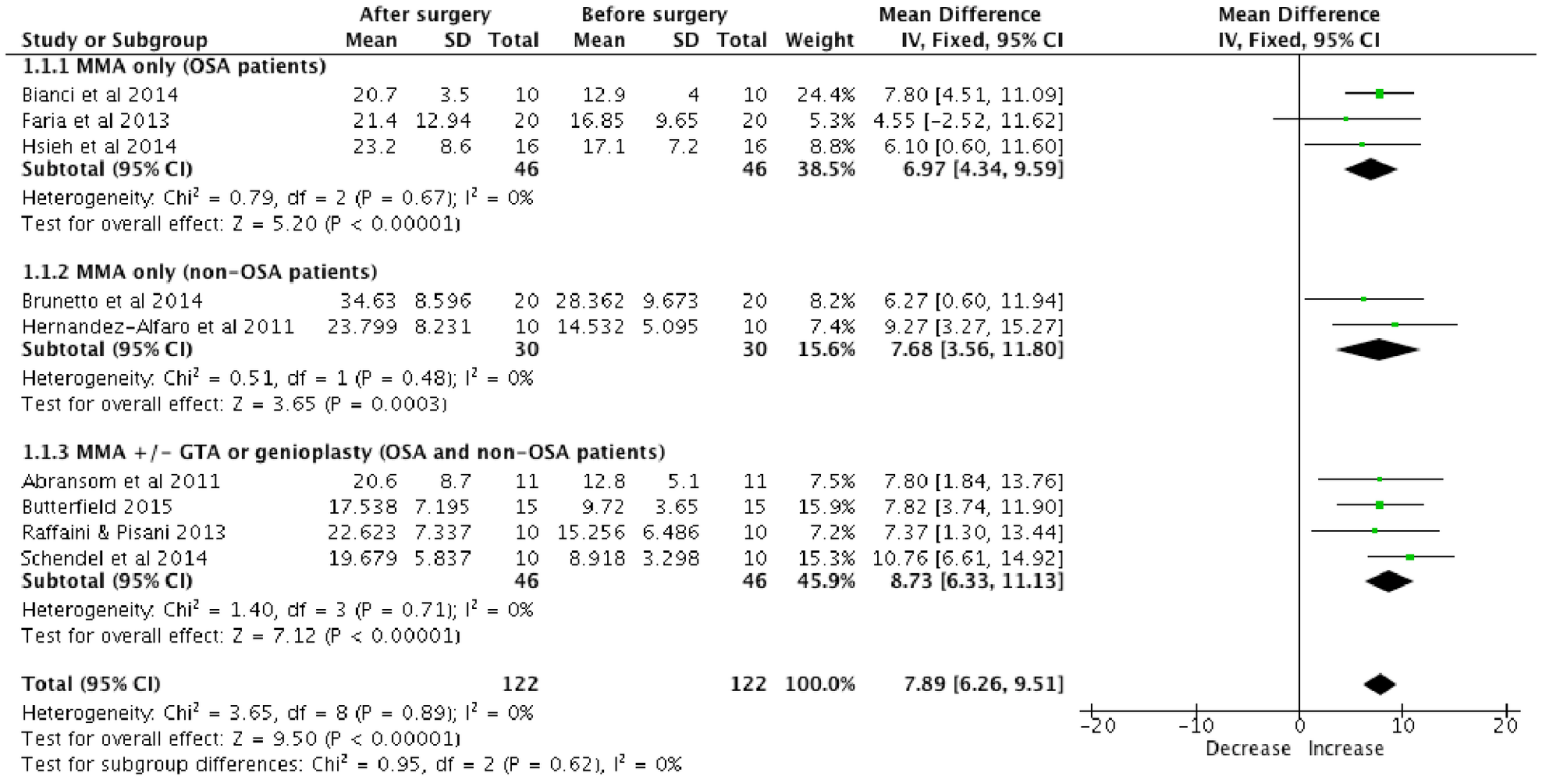
Forest plot of total volumetric changes of pharyngeal airway after MMA.

**Fig 3:**
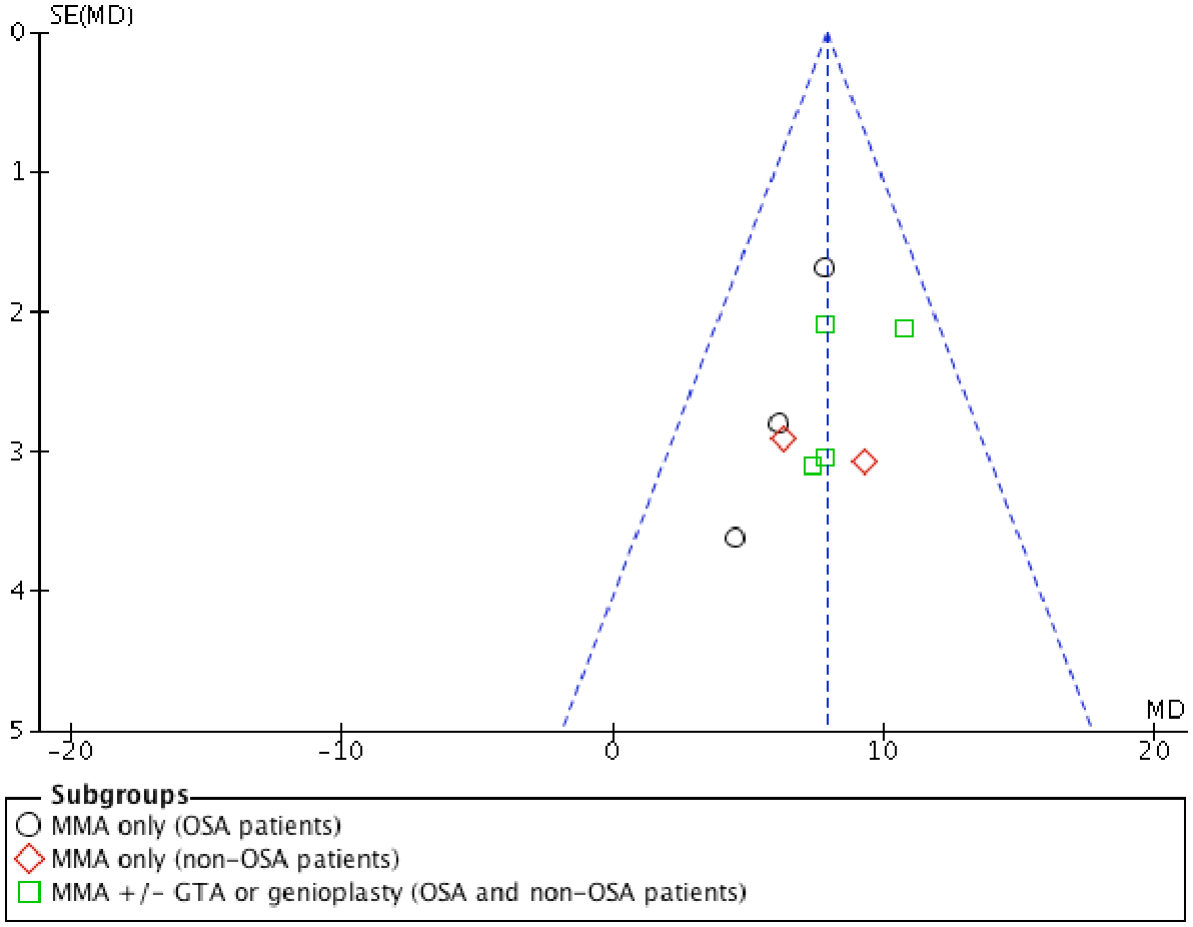
Funnel plot for MMA studies.

Meta-analysis of pharyngeal airway volumetric changes after MMA was performed by pooling the primary studies of Christovam *et al*[11] and Rosario *et al*[3] (Fig 2). As the heterogeneity was low (*I*^2^ = 0%), the fixed effect model was used. The meta-analysis indicated that MMA with or without genioplasty or genial tubercle advancement (GTA) lead to a significantly increased total pharyngeal volume (mean = 7.89ml; 95% CI 6.26, 9.51) after the surgery (p<0.00001). Although there was no statistically significant different (p = 0.62) between the subgroups, MMA with genioplasty or GTA has higher increased total pharyngeal volume (mean = 8.73ml) in comparison with MMA alone (mean = 6.97ml; 7.68ml) after the surgery. A symmetry funnel plot was noted suggesting a low risk of publication bias (Fig 3)

Two primary studies reported by Butterfield *et* al[42, 43] were found to have potentially overlapping participants. A confirmation attempt with the corresponding author has failed. Although Rosario *et al*[3]did not report any suspicion on this matter and have included both studies in their meta-analysis, only one[42] of the articles has been included for the meta-analysis in this overview to avoid potential duplication. Besides, another primary study[44] meta-analyzed by Rosario *et al*[3] was not included in this meta-analysis performed here because the maxillary procedures of that study comprised of those with or without advancement movement. On the other hand, two primary studies[45, 46] included but not meta-analyzed by Christovam *et al*[11] were found eligible to be included in the meta-analysis of this meta-analysis. However, one of these two studies[45]was eventually not included in this meta-analysis, as they have only reported mean value without standard deviation. The attempt to get further information from the corresponding author was not successful.

#### Respiratory parameters

Meta-analyses of post-MMA data reported by included systematic reviews in this overview revealed a significant reduction of the AHI[13, 18], Respiratory Disturbance Index (RDI)[18] and lowest nocturnal oxyhaemoglobin (SpO2 nadir) values[13] (Table 5). Two meta-analyses[12, 13] revealed similar results with high success rate. Another systematic review[18] further reported an 85.5% surgical success rate and a 38.5% cure rate.

Two systematic reviews[13, 18] have performed subgroup analyses based on pre-operative AHI of less than 30/h, 30 to 59.9/h, 60–89.9/h, 90 and above/h. Holty *et al*[13] reported AHI success rates of 81.0%, 88.5%, 81.2% and 80.4%, respectively, whereas Zaghi *et al*[18] described rates of 34%, 88%, 45% and 8%. The latter[18] also demonstrated that patients with higher pre-operative AHI experienced the biggest improvement, however, presenting the lowest chance to achieve the end points of surgical success and cure.

Hsieh and Liao[14] did not perform meta-analysis for their included 12 case series with 330 patients, but presented a mean success rate (AHI/RDI <20/h) of 87.03% (range: 65–100%). Two of their included primary case series did not report on their patients’ BMI (body mass index), while others provided mean values ranging from 22 to 45[14].

Univariate analysis of Holty *et al*[13] suggested that younger age (p = 0.013), lower pre-operative AHI (p = 0.027) and greater degree of maxillary advancement (p = 0.029) to be surgical success predictors. Their multivariate analysis further identified a lower pre-operative BMI as an additional surgery success predictor[13]. These results were supported by Zaghi *et al*[18] who have found younger age (p = 0.03), lower pre-operative AHI (p<0.001) and lower SpO_2_ nadir (p = 0.04) to be associated with a higher post-MAA OSA cure rate (AHI<5/h).

## Discussion

The here presented overview detected significantly reduced AHI after MMA with a relatively high treatment success rate (>85%) in OSA patients. This is comprehensible and in line with consistently increased post-MMA linear, cross-sectional area and volumetric pharyngeal airway measurements. The minimum CSA is one of the most commonly used airway measurements[9], and has been associated with the incidence of OSA[46]. A complete pharyngeal airway analysis should include linear, cross-sectional and volumetric analyses[14, 47] on various predefined areas to reveal the actual changes in all dimensions. Unfortunately, most articles did not assess all three aspects together. Additionally, to date, no specific guideline for standard assessment of pharyngeal airway evaluation exists, despite of its importance[11].

Mandibular advancement with bilateral sagittal split osteotomies (BSSO) is a well-established procedure in the treatment for patients with retrognathic mandible, with concomitant beneficial effect on pharyngeal airways.[4] However, vast majority of the included systematic reviews only focused on the effect of MMA onto pharyngeal airways and/or respiratory parameters. Although two reviews[11, 16] included a total of five primary studies[1, 2, 4–6] with isolated mandibular advancement osteotomies (BSSO), their findings and results were not elaborated in depth. This is most likely due to the small number and the heterogeneity of those primary studies. This overview of systematic reviews did not intend to study primary studies of included systematic reviews. Nevertheless, these five primary papers were retrieved and reported in this overview, yet without performing another electronic search for other primary articles of single-jaw mandibular advancement procedures. Those studies[1, 2, 4–6] reported significantly enlarged pharyngeal airway dimensions after isolated mandibular advancement osteotomies. However, this result was proved unstable during a long-term follow-up of 12 years, with lower parts of the pharyngeal airways relapsing to pre-operative values while upper and middle parts became significantly smaller than pre-operatively[4]. Future studies with longer follow-up periods are needed to verify those outcomes. Furthermore, still no evidence related to post-surgical pharyngeal airway changes after mandibular advancement combined with other concomitant maxillary osteotomies e.g. maxillary setback or maxillary impaction. Since those combined jaw movements are also commonly performed in orthognathic surgeries, future pharyngeal airway studies should also report on synergistic effects of those combined two jaws osteotomies.

Based on CBCT analysis, Hernandez-Alfaro *et al*[2]have reported that single-jaw mandibular advancement osteotomies lead to larger pharyngeal airway spaces (78.3%) in comparison with single-jaw maxillary advancement surgeries (37.7%). Interestingly, amore recent meta-analysis[13] of MMA considered the degree of maxillary instead of mandibular advancement to be a predictor of surgical success. Combined effects during two-jaw osteotomies might assert a different outcome on the attached musculature and soft tissue compared to single-jaw osteotomies. Two studies in the scope of MMA procedures for patients suffering from OSA stated that younger patients[13, 18] with lower pre-operative AHI[13, 18] and BMI[13]are associated with a higher surgery success[13] and OSA cure rate[18]. This clinical information would be helpful for surgeons in anticipating surgical outcome pre-surgically.

The maximum follow-up period varies across primary studies between 5 weeks to 12 years, with a vast majority of less than 5 years. Therefore, some of these follow-up periods were definitely too short since recurrence of OSA has been reported as late as 10 to 15 years after MMA[18]. A standardized period for long-term follow-up and the recording of pre-surgical BMI values in future studies might shall enhance the data comparison. Although some authors of primary studies have reported the amount of surgical jaw movements, many still neglected this important information in their report. As quite extensive evidence existed currently supporting the benefit of MMA on OSA patients, future studies should investigate the detailed correlations between pre-surgical clinical conditions, degree and direction of jaw movement and surgical success or cure rate. Nevertheless, other factor such as esthetic outcome after MMA especially in patients with normal pre-surgical skeletal pattern should also be assessed vigorously. These types of researches will generate valuable information for the pre-surgical planning to achieve optimum surgical and esthetic outcomes. An evidence-based clinical practical guideline with consideration of all those factors would probably the ultimate goal for MMA treatment in OSA patients.

Around one third of the included systematic reviews have performed electronic search in only one database and therefore posed a significant threat to selection bias. Moreover, none of the included systematic reviews has disclosed the ‘conflict of interest’ status of their included primary studies. Besides, four systematic reviews did not describe the characteristics of their included primary studies. As the quality of systematic reviews is affected directly by the quality of its included primary studies, a thorough investigation and reporting of each included study are mandatory.

Publication bias is another critical aspect to be investigated in the systematic reviews. Only two[16, 18] out of eight meta-analyses have assessed and reported the publication bias of their included primary studies. Christovam *et al*[11] suspected two groups of authors have reported on overlapping samples in different articles. However, their attempt to confirm with the authors has failed. Same issue came across during the process of this overview and ended up with same result too: attempt to contact the particular correspondent author was in vain. Beside the importance of avoiding duplicate publication, making a clear declaration for overlapping sample sizes in different papers is also very important to prevent future systematic reviews from reporting false results.

The following shortcomings of this overview have to be highlighted. Most primary studies of the included systematic reviews were of moderate and only a few of high quality, which might have affected the quality of those systematic reviews. Besides, seven of the included systematic reviews[9, 10, 12–15, 17] have fulfilled less than half of the AMSTAR criteria. Therefore, the results of this overview shall be read with caution.

## Conclusion

Maxillomandibular advancement (MMA) increases pharyngeal airway dimensions, providing positive post-surgical effects in patients suffering from OSA. However, still more evidence is needed to draw conclusions related to effect of single-jaw mandibular advancement osteotomies on pharyngeal airways.

## Supporting information

S1 Text Detailed search strategy. (PDF)

S2 Text Citation matrix. (PDF)

S1 Table PRISMA checklist. (PDF)
