# Supplementary material for: How does mandibular advancement with or without maxillary procedures affect pharyngeal airways? An overview of systematic reviews
Source: PLoS One. 2017 Jul 27;12(7):e0181146. doi: 10.1371/journal.pone.0181146 (PMC5531493; doi:10.1371/journal.pone.0181146)
Supplement: S1 Text — (PDF) [file pone.0181146.s001.pdf]

Appendix 1: Detailed search strategy

PUBMED

1. Review [Publication Type]
2. Review Literature as Topic [Mesh]
3. Meta-Analysis [Publication Type]
4. Meta-Analysis as Topic [Mesh]
5. Evidence-Based Medicine [Mesh]
6. Evidence-Based Dentistry [Mesh]
7. systematic review [all field]
8. review [all field]
9. overview [all field]
10. meta-analysis [all field]
11. evidence based medicine [all field]
12. evidence based dentistry [all field]
13. review literature [all field]
14. literature review [all field]
15. 1or 2 or 3 or 4 or 5 or 6 or 7 or 8 or 9 or 10 or 11 or 12 or 13 or 14
16. Orthognathic Surgery [Mesh]
17. Orthognathic Surgical Procedures [Mesh]
18. Mandibular advancement [Mesh]
19. orthognathic surgery [all field]
20. orthognathic surgical procedure [all field]
21. orthodontics surgery [all field]
22. maxillomandibular advancement [all field]
23. mandibular surgery [all field]
24. maxillary surgery [all field]
25. bimaxillary surgery [all field]
26. jaw surgery [all field]
27. surgical orthodontic treatment [all field]
28. jaw advancement [all field]
29. jaw movement [all field]
30. mandibular advancement [all field]
31. 16 or 17 or 18 or 19 or 20 or 21 or 22 or 23 or 24 or 25 or 26 or 27 or 28 or 29 or 30
32. Pharynx [Mesh]
33. upper airway [all field]
34. pharynx [all field]
35. pharyngeal [all field]
36. oropharynx [all field]
37. oropharyngeal [all field]
38. nasopharynx [all field]
39. nasopharyngeal [all field]
40. hypopharynx [all field]
41. hypopharyngeal [all field]
42. 32 or 33 or 34 or 35 or 36 or 37 or 38 or 39 or 40 or 41
43. 15 and 31 and 42

EMBASE

1. exp systematic review/
2. meta analysis/
3. exp evidence based medicine/
4. exp evidence based dentistry/
5. systematic review.mp.
6. review.mp.
7. overview.mp.
8. meta-analysis.mp.
9. evidence based medicine.mp.
10. evidence based dentistry.mp.
11. review literature.mp.
12. literature review.mp.
13. 1 or 2 or 3 or 4 or 5 or 6 or 7 or 8 or 9 or 10 or 11 or 12
14. exp orthognathic surgery/
15. orthognathic surgery.mp.
16. orthognathic surgical procedure.mp.
17. orthodontics surgery.mp.
18. maxillomandibular advancement.mp.
19. mandibular surgery.mp.
20. maxillary surgery.mp.
21. bimaxillary surgery.mp.
22. jaw surgery.mp.
23. surgical orthodontic treatment.mp.
24. jaw advancement.mp.
25. jaw movement.mp.
26. mandibular advancement.mp.
27. 14 or 15 or 16 or 17 or 18 or 19 or 20 or 21 or 22 or 23 or 24 or 25 or 26
28. exp pharynx/
29. upper airway.mp.
30. pharynx.mp.
31. pharyngeal.mp.
32. oropharynx.mp.
33. oropharyngeal.mp.
34. nasopharynx.mp.
35. nasopharyngeal.mp.
36. hypopharynx.mp.
37. hypopharyngeal.mp.
38. 28 or 29 or 30 or 31 or 32 or 33 or 34 or 35 or 36 or 37
39. 13 and 27 and 38

COCHRANE LIBRARY

1. MeSH descriptor: [Meta-Analysis] explode all trees
2. MeSH descriptor: [Review] explode all trees
3. MeSH descriptor: [Evidence-Based Medicine] explode all trees
4. MeSH descriptor: [Evidence-Based Dentistry] explode all trees
5. systematic review
6. review
7. overview
8. meta-analysis
9. evidence based medicine
10. evidence based dentistry
11. review literature
12. literature review
13. #1 or #2 or #3 or #4 or #5 or #6 or #7 or #8 or #9 or #10 or #11 or #12
14. MeSH descriptor: [Orthognathic Surgery] explode all trees
15. orthognathic surgery
16. orthognathic surgical procedure
17. orthodontics surgery
18. maxillomandibular advancement
19. mandibular surgery
20. maxillary surgery
21. bimaxillary surgery
22. jaw surgery
23. surgical orthodontic treatment
24. jaw advancement
25. jaw movement
26. mandibular advancement
27. #14 or #15 or #16 or #17 or #18 or #19 or #20 or #21 or #22 or #23 or #24 or #25 or #26
28. MeSH descriptor: [Pharynx] explode all trees
29. upper airway
30. pharynx
31. pharyngeal
32. oropharynx
33. oropharyngeal
34. hypopharynx
35. hypopharyngeal
36. #28 or #29 or #30 or #31 or #32 or #33 or #34 or #35
37. #13 and #27 and #36

SCOPUS

1. TITLE-ABS-KEY (systematic AND review)
2. TITLE-ABS-KEY (review)
3. TITLE-ABS-KEY (overview)
4. TITLE-ABS-KEY (meta-analysis)
5. TITLE-ABS-KEY (evidence AND based AND medicine)
6. TITLE-ABS-KEY (evidence AND based AND dentistry)
7. TITLE-ABS-KEY (review AND literature)
8. TITLE-ABS-KEY (literature AND review)
9. #1 or #2 or #3 or #4 or #5 or #6 or #7 or #8
10. TITLE-ABS-KEY (orthognathic AND surgery)
11. TITLE-ABS-KEY (orthognathic AND surgical AND procedure)
12. TITLE-ABS-KEY (orthodontics AND surgery)
13. TITLE-ABS-KEY (maxillomandibular AND advancement)
14. TITLE-ABS-KEY (mandibular AND surgery)
15. TITLE-ABS-KEY (maxillary AND surgery)
16. TITLE-ABS-KEY (bimaxillary AND surgery)
17. TITLE-ABS-KEY (jaw AND surgery)
18. TITLE-ABS-KEY (surgical AND orthodontic AND treatment)
19. TITLE-ABS-KEY (jaw AND advancement)
20. TITLE-ABS-KEY (jaw AND movement)
21. TITLE-ABS-KEY (mandibular AND advancement)
22. #10 or #11 or #12 or #13 or #14 or #15 or #16
23. #17 or #18 or #19 or #20 or #21 or #22
24. TITLE-ABS-KEY (upper AND airway)
25. TITLE-ABS-KEY (pharynx)
26. TITLE-ABS-KEY (pharyngeal)
27. TITLE-ABS-KEY (oropharyngeal)
28. TITLE-ABS-KEY (nasopharyngeal)
29. TITLE-ABS-KEY (hypopharyngeal)
30. TITLE-ABS-KEY (hypopharynx)
31. TITLE-ABS-KEY (nasopharynx)
32. TITLE-ABS-KEY (oropharynx)
33. #24 or #25 or #26 or #27 or #28 or #29 or #30
34. #31 or #32 or #33
35. #9 and #23 and #34

Web of Science
Search field: topic

(Systematic review OR review OR overview OR meta-analysis OR evidence based medicine OR review literature OR literature review) AND (orthognathic surgery OR orthognathic surgical procedure OR orthodontics surgery OR maxillomandibular advancement OR mandibular surgery OR maxillary surgery OR bimaxillary surgery OR jaw surgery OR surgical orthodontic treatment OR jaw advancement OR jaw movement OR mandibular advancement) AND (upper airway OR pharynx OR pharyngeal OR oropharynx OR oropharyngeal OR nasopharynx OR nasopharyngeal OR hypopharynx OR hypopharyngeal)
