# Supplementary material for: How does mandibular advancement with or without maxillary procedures affect pharyngeal airways? An overview of systematic reviews
Source: PLoS One. 2017 Jul 27;12(7):e0181146. doi: 10.1371/journal.pone.0181146 (PMC5531493; doi:10.1371/journal.pone.0181146)
Supplement: S2 Text — (PDF) [file pone.0181146.s002.pdf]

Appendix 2. Citation matrix

The citation matrices presented below have only included the primary papers that were analysed quantitatively in the four included meta-analyses of this overview to avoid false positive results. The following primary papers have not been included here:

- primary papers of systematic reviews without meta-analysis
- primary papers not quantitatively analysed in systematic review with meta-analysis

The corrected covered area (CCA)[1] was calculated separately for different measurement analyses based on its own citation matrix to represent the actual overlapping situation. When the measurement only reported by one systematic review, then CCA will not be calculated.

Formula of CCA[1]: CCA= (N-r)/(rc-r)

N= number of included publications (including double counting)
r= number of rows (number of index publications)
c= number of columns (number of reviews)

Interpretation of CCA scores[1]:

0-5 : slight overlap
6-10: moderate overlap
11-15: high overlap
>15: very high overlap

Table 1. Citation matrix of primary papers related to antero-posterior dimension measurement of pharyngeal airway at the level of soft palate

		**Mattos *et al*, 2011[2]**
Primary papers	Goncalves *et al*, 2006[3]	/
	Mehra *et al*, 2001[4]	/

Table 2. Citation matrix of primary papers related to minimum cross-sectional measurement of pharyngeal airway

		**Christovam *et al*, 2015[5]**
Primary papers	Abransom *et al*, 2011[6]	/
	Raffaini & Pisani, 2013[7]	/
	Schendel *et al*, 2014[8]	/

Table 3. Citation matrix of primary papers related to total volumetric changes of pharyngeal airway

		**Christovam *et al*, 2015[5]**	**Rosario *et al*, 2016[9]**
Primary papers	Bianchi *et al*, 2014[10]	/	
	Brunetto *et al*, 2014[11]	/	
	Hernandez-Alfrado *et al*, 2011[12]	/	/
	Hsieh *et al*, 2014[13]	/	
	Raffaini & Pisani, 2013[7]	/	/
	Abransom *et al*, 2011[6]		/
	Butterfield *et al*, 2015a[14]		/
	Butterfield *et al*, 2015b[15]		/
	Valladeres-Neto *et al*, 2013[16]		/

CCA= 22% (very high overlap)

Table 4. Citation matrix of surgical success rates after MMA

		**Holty *et al*, 2010[17]**	**Elshaug *et al*, 2007[18]**
Primary studies	Yu et al, 2009[19]	/	
	Lye et al, 2005[20]	/	
	Lye et al, 2007[21]	/	
	Lu et al, 2007[22]	/	
	Fairbun et al, 2007[23]	/	
	Dekeister et al, 2006[24]	/	
	Hoekema et al, 2006[25]	/	
	Smatt & Ferri, 2005[26]	/	
	Dattillo & Drooger, 2004[27]	/	/
	Goh & Lim, 2003[28]	/	/
	Li et al, 2002[29]	/	/
	Hendler et al, 2001[30]	/	/
	Li et al, 2000-1[31] [32-34]	/	
	Bettega et al, 2000[35]	/	
	Gregg et al, 2000[36]	/	
	Wagner et al, 2000[37, 38]	/	
	Li et al, 2000-3[39-41]	/	
	Lee et al, 1999[42]	/	
	Prinsell 1999[43]	/	
	Hochban et al, 1994-7[44-47]	/	
	Waite et al, 1989[48]	/	
	Riley et al, 1986[49]	/	

CCA= 18.2% (very high overlap)

Table 5. Citation matrix of surgical cure rates after MMA

		**Holty *et al*, 2010[17]**
Primary studies	Yu et al, 2009[19]	/
	Lu et al, 2007[22]	/
	Fairbun et al, 2007[23]	/
	Dekeister et al, 2006[24]	/
	Hoekema et al, 2006[25]	/
	Smatt & Ferri, 2005[26]	/
	Dattillo & Drooger, 2004[27]	/
	Goh & Lim, 2003[28]	/
	Hendler et al, 2001[30]	/
	Li et al, 2000-1[31] [32-34]	/
	Wagner et al, 2000[37, 38]	/
	Li et al, 2000-3[39-41]	/
	Lee et al, 1999[42]	/
	Hochban et al, 1994-7[44-47]	/
	Waite et al, 1989[48]	/
	Riley et al, 1986[49]	/

Table 6. Citation matrix of apnea-hypopnea index (AHI) outcomes after MMA

		**Zaghi *et al*, 2015[50]**	**Holty *et al*, 2010[17]**	**Caples *et al*, 2010[51]**	**Knudsen *et al*, 2015[52]**
Primary studies	Serra *et al*, 2012[53]	/			
	Brevi *et al*, 2011[54]	/			
	Riley *et al*, 1990[55]	/		/	
	Vicini *et al*, 2010[56]	/			
	Riley *et al*, 1989[57]	/			
	Jalbert *et al*, 2012[58]	/			
	Varghese *et al*, 2012[59]	/			
	Li *et al*, 2000[34]	/			
	Hochban *et al*, 1994[44]	/			
	Hochban *et al*, 1997[45]	/			
	Ronchi *et al*, 2010[60]	/			
	Fairbun *et al*, 2007[23]	/	/		/
	Li *et al*, 2000[40]	/			
	Wagner *et al*, 2000[37]	/			
	Ronchi *et al*, 2013[61]	/			
	Riley *et al*, 1990[62]	/			
	Hochban *et al*, 1997[44]	/		/	
	Lin *et al*, 2011[63]	/			/
	Goh & Lim, 2003[28]	/			
	Schendel *et al*, 2014[8]	/			
	Waite *et al*, 1989[48]	/			
	Riley *et al*, 1986[49]	/			
	Li *et al*, 2010[64]	/			
	Jaspers *et al*, 2013[65]	/			
	de Lange *et al*, 2004[66]	/			
	Hoekema *et al*, 2006[25]	/			
	Barrera 2013[67]	/			
	Yu *et al*, 2009[19]	/			
	Sencimen *et al*, 2012[68]	/			
	Schendel *et al*, 2011[69]	/			
	Matsuo *et al*, 2009[70]	/			
	Johnson & Boyd, 2002[71]	/			
	El *et al*, 2011[72]	/			
	Doff *et al*, 2013[73]	/			
	Corcoran *et al*, 2009[74]	/			
	Barrera *et al*, 2007[75]	/			
	Banhiran *et al*, 2007[76]	/			
	Yu *et al*, 2009[19]		/		
	Lye *et al*, 2008[20, 21]		/		
	Lu *et al*, 2007[22]		/		
	Dekeister *et al*, 2006[24]		/	/	
	Hoekema *et al*, 2006[25]		/		

	Smatt & Ferri, 2005[26]		/		
	Dattillo & Drooger, 2004[27]		/		
	Goh & Lim, 2003[28]		/	/	
	Li *et al*, 2002[29]		/		
	Hendler *et al*, 2001[30]		/		
	Li *et al*, 2000-1[31] [32-34]		/		
	Bettega *et al*, 2000[35]		/		
	Gregg *et al*, 2000[36]		/	/	
	Wagner et al, 2000[37, 38]		/		
	Li et al, 2000-3[39-41]		/		
	Lee et al, 1999[42]		/		
	Prinsell 1999[43]		/	/	
	Conradt et al, 1996[77]		/		
	Conradt et al, 1998[78]		/	/	
	Conradt et al, 1997[46]			/	
	Hochban et al, 1994-7[44-47]		/		
	Waite et al, 1989[48]		/		
	Riley et al, 1986[49]		/		
	Kessler et al, 2007[79]			/	
	Abransom et al, 2011[6]				/
	Zinser et al, 2013[80]				/

CCA=5.29%(slight overlap)

Table 7. Citation matrix of respiratory disturbance index (RDI) outcomes after MMA

		**Zaghi *et al*, 2015[50]**
Primary studies	Jones *et al*, 2010[81]	/
	Dattilo & Drooger, 2004[27]	/
	Arcuri *et al*, 2011[82]	/
	Hendler *et al*, 2001[30]	/
	Abransom *et al*, 2011[6]	/
	Barrera 2013[67]	/
	Lee *et al*, 1999[42]	/
	Van Sickels &Wallender, 2012[83]	/
	Conley & Legan, 2006[84]	/
	Colin, 2004[85]	/
	Banhiran *et al*, 2007[76]	/

References:

1. Pieper D, Antoine S-L, Mathes T, Neugebauer EAM, Eikermann M. Systematic review finds overlapping reviews were not mentioned in every other overview. Journal of clinical epidemiology. 2014;67:368-75.
2. Mattos CT, Vilani GN, Sant'Anna EF, Ruellas AC, Maia LC. Effects of orthognathic surgery on oropharyngeal airway: a meta-analysis. Int J Oral Maxillofac Surg. 2011;40(12):1347-56. Epub 2011/07/26. doi: 10.1016/j.ijom.2011.06.020. PubMed PMID: 21782388.
3. Goncalves JR, Buschang PH, Goncalves DG, Wolford LM. Postsurgical stability of oropharyngeal airway changes following counter-clockwise maxillo-mandibular advancement surgery. Journal of oral and maxillofacial surgery : official journal of the American Association of Oral and Maxillofacial Surgeons. 2006;64(5):755-62. Epub 2006/04/25. doi: 10.1016/j.joms.2005.11.046. PubMed PMID: 16631481.
4. Mehra P, Downie M, Pita MC, Wolford LM. Pharyngeal airway space changes after counterclockwise rotation of the maxillomandibular complex. American journal of orthodontics and dentofacial orthopedics : official publication of the American Association of Orthodontists, its constituent societies, and the American Board of Orthodontics. 2001;120(2):154-9. Epub 2001/08/14. doi: 10.1067/mod.2001.114647. PubMed PMID: 11500657.
5. Christovam IO, Lisboa CO, Ferreira D, Cury-Saramago AA, Mattos CT. Upper airway dimensions in patients undergoing orthognathic surgery: a systematic review and meta-analysis. International Journal of Oral and Maxillofacial Surgery. 2016;45(4):460-71. doi: 10.1016/j.ijom.2015.10.018. PubMed PMID: WOS:000372938300008.
6. Abramson Z, Susarla SM, Lawler M, Bouchard C, Troulis M, Kaban LB. Three-dimensional computed tomographic airway analysis of patients with obstructive sleep apnea treated by maxillomandibular advancement. Journal of oral and maxillofacial surgery : official journal of the American Association of Oral and Maxillofacial Surgeons. 2011;69(3):677-86. Epub 2011/03/01. doi: 10.1016/j.joms.2010.11.037. PubMed PMID: 21353929.
7. Raffaini M, Pisani C. Clinical and cone-beam computed tomography evaluation of the three-dimensional increase in pharyngeal airway space following maxillo-mandibular rotation-advancement for Class II-correction in patients without sleep apnoea (OSA). Journal of cranio-maxillo-facial surgery : official publication of the European Association for Cranio-Maxillo-Facial Surgery. 2013;41(7):552-7. Epub 2013/01/15. doi: 10.1016/j.jcms.2012.11.022. PubMed PMID: 23312953.
8. Schendel SA, Broujerdi JA, Jacobson RL. Three-dimensional upper-airway changes with maxillomandibular advancement for obstructive sleep apnea treatment. American journal of orthodontics and dentofacial orthopedics : official publication of the American Association of Orthodontists, its constituent societies, and the American Board of Orthodontics. 2014;146(3):385-93. Epub 2014/08/31. doi: 10.1016/j.ajodo.2014.01.026. PubMed PMID: 25172261.
9. Rosario HD, Oliveira GM, Freires IA, de Souza Matos F, Paranhos LR. Efficiency of bimaxillary advancement surgery in increasing the volume of the upper airways: a systematic review of observational studies and meta-analysis. European archives of oto-rhino-laryngology : official journal of the European Federation of Oto-Rhino-Laryngological Societies (EUFOS) : affiliated with the German Society for Oto-Rhino-Laryngology - Head and Neck Surgery. 2016. Epub 2016/03/31. doi: 10.1007/s00405-016-4015-4. PubMed PMID: 27028016.
10. Bianchi A, Betti E, Tarsitano A, Morselli-Labate AM, Lancellotti L, Marchetti C. Volumetric three-dimensional computed tomographic evaluation of the upper airway in patients with obstructive sleep apnoea syndrome treated by maxillomandibular advancement. The British journal of oral & maxillofacial surgery. 2014;52(9):831-7. Epub 2014/08/19. doi: 10.1016/j.bjoms.2014.07.101. PubMed PMID: 25129655.
11. Brunetto DP, Velasco L, Koerich L, Araujo MT. Prediction of 3-dimensional pharyngeal airway changes after orthognathic surgery: a preliminary study. American journal of orthodontics and dentofacial orthopedics : official publication of the American Association of Orthodontists, its constituent societies, and the American Board of Orthodontics. 2014;146(3):299-309. Epub 2014/08/31. doi: 10.1016/j.ajodo.2014.05.024. PubMed PMID: 25172252.
12. Hernandez-Alfaro F, Guijarro-Martinez R, Mareque-Bueno J. Effect of mono- and bimaxillary advancement on pharyngeal airway volume: cone-beam computed tomography evaluation. Journal of oral and maxillofacial surgery : official journal of the American Association of Oral and Maxillofacial

Surgeons. 2011;69(11):e395-400. Epub 2011/07/30. doi: 10.1016/j.joms.2011.02.138. PubMed PMID: 21798648.
13. Hsieh YJ, Liao YF, Chen NH, Chen YR. Changes in the calibre of the upper airway and the surrounding structures after maxillomandibular advancement for obstructive sleep apnoea. The British journal of oral & maxillofacial surgery. 2014;52(5):445-51. Epub 2014/03/19. doi: 10.1016/j.bjoms.2014.02.006. PubMed PMID: 24629456.
14. Butterfield KJ, Marks PL, McLean L, Newton J. Linear and volumetric airway changes after maxillomandibular advancement for obstructive sleep apnea. Journal of oral and maxillofacial surgery : official journal of the American Association of Oral and Maxillofacial Surgeons. 2015;73(6):1133-42. Epub 2015/03/22. doi: 10.1016/j.joms.2014.11.020. PubMed PMID: 25795186.
15. Butterfield KJ, Marks PL, McLean L, Newton J. Pharyngeal airway morphology in healthy individuals and in obstructive sleep apnea patients treated with maxillomandibular advancement: a comparative study. Oral surgery, oral medicine, oral pathology and oral radiology. 2015;119(3):285-92. Epub 2015/01/17. doi: 10.1016/j.oooo.2014.11.016. PubMed PMID: 25592868.
16. Valladares-Neto J, Silva MA, Bumann A, Paiva JB, Rino-Neto J. Effects of mandibular advancement surgery combined with minimal maxillary displacement on the volume and most restricted cross-sectional area of the pharyngeal airway. Int J Oral Maxillofac Surg. 2013;42(11):1437-45. Epub 2013/05/25. doi: 10.1016/j.ijom.2013.03.018. PubMed PMID: 23702371.
17. Holty JE, Guilleminault C. Maxillomandibular advancement for the treatment of obstructive sleep apnea: a systematic review and meta-analysis. Sleep medicine reviews. 2010;14(5):287-97. Epub 2010/03/02. doi: 10.1016/j.smrv.2009.11.003. PubMed PMID: 20189852.
18. Elshaug AG, Moss JR, Southcott AM, Hiller JE. Redefining success in airway surgery for obstructive sleep apnea: a meta analysis and synthesis of the evidence. Sleep. 2007;30(4):461-7. Epub 2007/05/25. PubMed PMID: 17520790.
19. Yu CC, Hsiao HD, Lee LC, Yao CM, Chen NH, Wang CJ, et al. Computational fluid dynamic study on obstructive sleep apnea syndrome treated with maxillomandibular advancement. The Journal of craniofacial surgery. 2009;20(2):426-30. Epub 2009/03/24. doi: 10.1097/SCS.0b013e31819b9671. PubMed PMID: 19305244.
20. Lye KW, Waite PD, Meara D, Wang D. Quality of life evaluation of maxillomandibular advancement surgery for treatment of obstructive sleep apnea. Journal of oral and maxillofacial surgery : official journal of the American Association of Oral and Maxillofacial Surgeons. 2008;66(5):968-72. Epub 2008/04/22. doi: 10.1016/j.joms.2007.11.031. PubMed PMID: 18423288.
21. Lye KW, Waite PD, Wang D, Sittitavornwong S. Predictability of prebent advancement plates for use in maxillomandibular advancement surgery. Journal of oral and maxillofacial surgery : official journal of the American Association of Oral and Maxillofacial Surgeons. 2008;66(8):1625-9. Epub 2008/07/19. doi: 10.1016/j.joms.2007.11.034. PubMed PMID: 18634950.
22. Lu XF, Zhu M, He JD, Zhang R, Li ZY, Sun HX. [Uvulopalatopharyngoplasty and maxillomandibular advancement for obese patients with obstructive sleep apnea hypopnea syndrome: a preliminary report]. Zhonghua kou qiang yi xue za zhi = Zhonghua kouqiang yixue zazhi = Chinese journal of stomatology. 2007;42(4):199-202. Epub 2007/08/09. PubMed PMID: 17683662.
23. Fairburn SC, Waite PD, Vilos G, Harding SM, Bernreuter W, Cure J, et al. Three-dimensional changes in upper airways of patients with obstructive sleep apnea following maxillomandibular advancement. Journal of oral and maxillofacial surgery : official journal of the American Association of Oral and Maxillofacial Surgeons. 2007;65(1):6-12. Epub 2006/12/19. doi: 10.1016/j.joms.2005.11.119. PubMed PMID: 17174756.
24. Dekeister C, Lacassagne L, Tiberge M, Montemayor T, Migueres M, Paoli JR. [Mandibular advancement surgery in patients with severe obstructive sleep apnea uncontrolled by continuous positive airway pressure. A retrospective review of 25 patients between 1998 and 2004]. Revue des maladies respiratoires. 2006;23(5 Pt 1):430-7. Epub 2007/02/23. PubMed PMID: 17314742.
25. Hoekema A, de Lange J, Stegenga B, de Bont LG. Oral appliances and maxillomandibular advancement surgery: an alternative treatment protocol for the obstructive sleep apnea-hypopnea syndrome. Journal of oral and maxillofacial surgery : official journal of the American Association of Oral and Maxillofacial Surgeons. 2006;64(6):886-91. Epub 2006/05/23. doi: 10.1016/j.joms.2005.11.041. PubMed PMID: 16713801.
26. Smatt Y, Ferri J. Retrospective study of 18 patients treated by maxillomandibular advancement with adjunctive procedures for obstructive sleep apnea syndrome. The Journal of craniofacial surgery. 2005;16(5):770-7. Epub 2005/09/30. PubMed PMID: 16192855.

27. Dattilo DJ, Drooger SA. Outcome assessment of patients undergoing maxillofacial procedures for the treatment of sleep apnea: comparison of subjective and objective results. Journal of oral and maxillofacial surgery : official journal of the American Association of Oral and Maxillofacial Surgeons. 2004;62(2):164-8. Epub 2004/02/06. PubMed PMID: 14762748.
28. Goh YH, Lim KA. Modified maxillomandibular advancement for the treatment of obstructive sleep apnea: a preliminary report. Laryngoscope. 2003;113(9):1577-82. Epub 2003/09/16. doi: 10.1097/00005537-200309000-00031. PubMed PMID: 12972937.
29. Li KK, Guilleminault C, Riley RW, Powell NB. Obstructive sleep apnea and maxillomandibular advancement: an assessment of airway changes using radiographic and nasopharyngoscopic examinations. Journal of oral and maxillofacial surgery : official journal of the American Association of Oral and Maxillofacial Surgeons. 2002;60(5):526-30; discussion 31. Epub 2002/05/04. PubMed PMID: 11988930.
30. Hendler BH, Costello BJ, Silverstein K, Yen D, Goldberg A. A protocol for uvulopalatopharyngoplasty, mortised genioplasty, and maxillomandibular advancement in patients with obstructive sleep apnea: an analysis of 40 cases. Journal of oral and maxillofacial surgery : official journal of the American Association of Oral and Maxillofacial Surgeons. 2001;59(8):892-7; discussion 8-9. Epub 2001/07/28. doi: 10.1053/joms.2001.25275. PubMed PMID: 11474445.
31. Li KK, Troell RJ, Riley RW, Powell NB, Koester U, Guilleminault C. Uvulopalatopharyngoplasty, maxillomandibular advancement, and the velopharynx. Laryngoscope. 2001;111(6):1075-8. Epub 2001/06/19. doi: 10.1097/00005537-200106000-00027. PubMed PMID: 11404624.
32. Li KK, Riley RW, Powell NB, Guilleminault C. Patient's perception of the facial appearance after maxillomandibular advancement for obstructive sleep apnea syndrome. Journal of oral and maxillofacial surgery : official journal of the American Association of Oral and Maxillofacial Surgeons. 2001;59(4):377-80; discussion 80-1. Epub 2001/04/06. doi: 10.1053/joms.2001.21870. PubMed PMID: 11289166.
33. Li KK, Riley RW, Powell NB, Gervacio L, Troell RJ, Guilleminault C. Obstructive sleep apnea surgery: patient perspective and polysomnographic results. Otolaryngology--head and neck surgery : official journal of American Academy of Otolaryngology-Head and Neck Surgery. 2000;123(5):572-5. Epub 2000/11/15. doi: 10.1067/mhn.2000.110107. PubMed PMID: 11077343.
34. Li KK, Riley RW, Powell NB, Guilleminault C. Maxillomandibular advancement for persistent obstructive sleep apnea after phase I surgery in patients without maxillomandibular deficiency. Laryngoscope. 2000;110(10 Pt 1):1684-8. Epub 2000/10/19. doi: 10.1097/00005537-200010000-00021. PubMed PMID: 11037825.
35. Bettega G, Pepin JL, Veale D, Deschaux C, Raphael B, Levy P. Obstructive sleep apnea syndrome. fifty-one consecutive patients treated by maxillofacial surgery. American journal of respiratory and critical care medicine. 2000;162(2 Pt 1):641-9. Epub 2000/08/10. doi: 10.1164/ajrccm.162.2.9904058. PubMed PMID: 10934100.
36. Gregg JM, Zedalis D, Howard CW, Boyle RP, Prussin AJ. Surgical alternatives for treatment of obstructive sleep apnoea: review and case series. Annals of the Royal Australasian College of Dental Surgeons. 2000;15:181-4. Epub 2001/11/17. PubMed PMID: 11709935.
37. Wagner I, Coiffier T, Sequert C, Lachiver X, Fleury B, Chabolle F. [Surgical treatment of severe sleep apnea syndrome by maxillomandibular advancing or mental tranposition]. Annales d'oto-laryngologie et de chirurgie cervico faciale : bulletin de la Societe d'oto-laryngologie des hopitaux de Paris. 2000;117(3):137-46. Epub 2000/06/23. PubMed PMID: 10863198.
38. De Dieuleveult T, Wagner I, Meulien P, Fleury B, Hausser-Hawn C, Chabolle F. [Retrospective cephalometric analysis for surgically treated obstructive sleep apnea: therapeutic deductions]. Annales d'oto-laryngologie et de chirurgie cervico faciale : bulletin de la Societe d'oto-laryngologie des hopitaux de Paris. 2000;117(6):339-48. Epub 2001/01/10. PubMed PMID: 11148338.
39. Li KK. Surgical management of obstructive sleep apnea. Clinics in chest medicine. 2003;24(2):365-70. Epub 2003/06/13. PubMed PMID: 12800790.
40. Li KK, Powell NB, Riley RW, Zonato A, Gervacio L, Guilleminault C. Morbidly obese patients with severe obstructive sleep apnea: is airway reconstructive surgery a viable treatment option? Laryngoscope. 2000;110(6):982-7. Epub 2000/06/14. doi: 10.1097/00005537-200006000-00019. PubMed PMID: 10852518.
41. Li KK, Powell NB, Riley RW, Troell RJ, Guilleminault C. Long-Term Results of Maxillomandibular Advancement Surgery. Sleep & breathing = Schlaf & Atmung. 2000;4(3):137-40. Epub 2002/02/28. doi: 10.1007/s11325-000-0137-3. PubMed PMID: 11868133.

42. Lee NR, Givens CD, Jr., Wilson J, Robins RB. Staged surgical treatment of obstructive sleep apnea syndrome: a review of 35 patients. Journal of oral and maxillofacial surgery : official journal of the American Association of Oral and Maxillofacial Surgeons. 1999;57(4):382-5. Epub 1999/04/13. PubMed PMID: 10199488.
43. Prinsell JR. Maxillomandibular advancement surgery in a site-specific treatment approach for obstructive sleep apnea in 50 consecutive patients. Chest. 1999;116(6):1519-29. Epub 1999/12/14. PubMed PMID: 10593771.
44. Hochban W, Conradt R, Brandenburg U, Heitmann J, Peter JH. Surgical maxillofacial treatment of obstructive sleep apnea. Plastic and reconstructive surgery. 1997;99(3):619-26; discussion 27-8. Epub 1997/03/01. PubMed PMID: 9047179.
45. Hochban W, Brandenburg U, Peter JH. Surgical treatment of obstructive sleep apnea by maxillomandibular advancement. Sleep. 1994;17(7):624-9. Epub 1994/10/01. PubMed PMID: 7846461.
46. Conradt R, Hochban W, Brandenburg U, Heitmann J, Peter JH. Long-term follow-up after surgical treatment of obstructive sleep apnoea by maxillomandibular advancement. The European respiratory journal. 1997;10(1):123-8. Epub 1997/01/01. PubMed PMID: 9032503.
47. Hochban W, Brandenburg U, Peter JH. [Surgical treatment of obstructive sleep apnea by osteotomy of the facial bones (results of 1 year)]. Pneumologie (Stuttgart, Germany). 1995;49 Suppl 1:175-9. Epub 1995/03/01. PubMed PMID: 7617608.
48. Waite PD, Wooten V, Lachner J, Guyette RF. Maxillomandibular advancement surgery in 23 patients with obstructive sleep apnea syndrome. Journal of oral and maxillofacial surgery : official journal of the American Association of Oral and Maxillofacial Surgeons. 1989;47(12):1256-61; discussion 62. Epub 1989/12/01. PubMed PMID: 2585177.
49. Riley RW, Powell NB, Guilleminault C, Nino-Murcia G. Maxillary, mandibular, and hyoid advancement: an alternative to tracheostomy in obstructive sleep apnea syndrome. Otolaryngology--head and neck surgery : official journal of American Academy of Otolaryngology-Head and Neck Surgery. 1986;94(5):584-8. Epub 1986/06/01. doi: 10.1177/019459988609400509. PubMed PMID: 3088520.
50. Zaghi S, Holty JE, Certal V, Abdullatif J, Guilleminault C, Powell NB, et al. Maxillomandibular Advancement for Treatment of Obstructive Sleep Apnea: A Meta-analysis. JAMA otolaryngology--head & neck surgery. 2016;142(1):58-66. Epub 2015/11/26. doi: 10.1001/jamaoto.2015.2678. PubMed PMID: 26606321.
51. Caples SM, Rowley JA, Prinsell JR, Pallanch JF, Elamin MB, Katz SG, et al. Surgical modifications of the upper airway for obstructive sleep apnea in adults: a systematic review and meta-analysis. Sleep. 2010;33(10):1396-407. Epub 2010/11/11. PubMed PMID: 21061863; PubMed Central PMCID: PMCPMC2941427.
52. Knudsen TB, Laulund AS, Ingerslev J, Homoe P, Pinholt EM. Improved apnea-hypopnea index and lowest oxygen saturation after maxillomandibular advancement with or without counterclockwise rotation in patients with obstructive sleep apnea: a meta-analysis. Journal of oral and maxillofacial surgery : official journal of the American Association of Oral and Maxillofacial Surgeons. 2015;73(4):719-26. Epub 2014/12/03. doi: 10.1016/j.joms.2014.08.006. PubMed PMID: 25443377.
53. Serra MM, Greenburg D, Barnwell M, Fallah D, Keith K, Mysliwiec V. Maxillomandibular advancement as surgical treatment for obstructive sleep apnea in active duty military personnel: a retrospective cohort. Military medicine. 2012;177(11):1387-92. Epub 2012/12/04. PubMed PMID: 23198518.
54. Brevi BC, Toma L, Pau M, Sesenna E. Counterclockwise rotation of the occlusal plane in the treatment of obstructive sleep apnea syndrome. Journal of oral and maxillofacial surgery : official journal of the American Association of Oral and Maxillofacial Surgeons. 2011;69(3):917-23. Epub 2011/01/11. doi: 10.1016/j.joms.2010.06.189. PubMed PMID: 21216064.
55. Riley RW, Powell NB, Guilleminault C. Maxillofacial surgery and nasal CPAP. A comparison of treatment for obstructive sleep apnea syndrome. Chest. 1990;98(6):1421-5. Epub 1990/12/01. PubMed PMID: 2245683.
56. Vicini C, Dallan I, Campanini A, De Vito A, Barbanti F, Giorgiomarrano G, et al. Surgery vs ventilation in adult severe obstructive sleep apnea syndrome. American journal of otolaryngology. 2010;31(1):14-20. Epub 2009/12/01. doi: 10.1016/j.amjoto.2008.09.002. PubMed PMID: 19944893.

57. Riley RW, Powell NB, Guilleminault C. Maxillofacial surgery and obstructive sleep apnea: a review of 80 patients. Otolaryngology--head and neck surgery : official journal of American Academy of Otolaryngology-Head and Neck Surgery. 1989;101(3):353-61. Epub 1989/09/01. doi: 10.1177/019459988910100309. PubMed PMID: 2508006.
58. Jalbert F, Lacassagne L, Bessard J, Dekeister C, Paoli JR, Tiberge M. [Oral appliances or maxillomandibular advancement osteotomy for severe obstructive sleep apnoea in patients refusing CPAP]. Revue de stomatologie et de chirurgie maxillo-faciale. 2012;113(1):19-26. Epub 2012/01/17. doi: 10.1016/j.stomax.2011.11.005. PubMed PMID: 22244739.
59. Varghese R, Adams NG, Slocumb NL, Viozzi CF, Ramar K, Olson EJ. Maxillomandibular advancement in the management of obstructive sleep apnea. International journal of otolaryngology. 2012;2012:373025. Epub 2012/04/21. doi: 10.1155/2012/373025. PubMed PMID: 22518154; PubMed Central PMCID: PMCPMC3299305.
60. Ronchi P, Novelli G, Colombo L, Valsecchi S, Oldani A, Zucconi M, et al. Effectiveness of maxillo-mandibular advancement in obstructive sleep apnea patients with and without skeletal anomalies. Int J Oral Maxillofac Surg. 2010;39(6):541-7. Epub 2010/05/04. doi: 10.1016/j.ijom.2010.03.006. PubMed PMID: 20434311.
61. Ronchi P, Cinquini V, Ambrosoli A, Caprioglio A. Maxillomandibular advancement in obstructive sleep apnea syndrome patients: a restrospective study on the sagittal cephalometric variables. Journal of oral & maxillofacial research. 2013;4(2):e5. Epub 2014/01/15. doi: 10.5037/jomr.2013.4205. PubMed PMID: 24422033; PubMed Central PMCID: PMCPMC3886110.
62. Riley RW, Powell NB, Guilleminault C. Maxillary, mandibular, and hyoid advancement for treatment of obstructive sleep apnea: a review of 40 patients. Journal of oral and maxillofacial surgery : official journal of the American Association of Oral and Maxillofacial Surgeons. 1990;48(1):20-6. Epub 1990/01/01. PubMed PMID: 2294208.
63. Lin CH, Liao YF, Chen NH, Lo LJ, Chen YR. Three-dimensional computed tomography in obstructive sleep apneics treated by maxillomandibular advancement. Laryngoscope. 2011;121(6):1336-47. Epub 2011/05/11. doi: 10.1002/lary.21813. PubMed PMID: 21557248.
64. Li Y, Yi B, Wang X, Li ZL, Liang C, Wang XX. [Clinical study on modified maxillomandibular advancement for the treatment of obstructive sleep apnea syndrome]. Beijing da xue xue bao Yi xue ban = Journal of Peking University Health sciences. 2010;42(5):570-4. Epub 2010/10/20. PubMed PMID: 20957017.
65. Jaspers GW, Booij A, de Graaf J, de Lange J. Long-term results of maxillomandibular advancement surgery in patients with obstructive sleep apnoea syndrome. The British journal of oral & maxillofacial surgery. 2013;51(3):e37-9. Epub 2012/05/09. doi: 10.1016/j.bjoms.2012.03.013. PubMed PMID: 22560785.
66. de Lange J, de Graaf J, Veldhuijzen van Zanten L, Waalkens HA. [Treatment of snoring and sleep apnea. Maxillo-mandibular advancement osteotomy]. Nederlands tijdschrift voor tandheelkunde. 2004;111(7):287-90. Epub 2004/08/19. PubMed PMID: 15315109.
67. Barrera JE. Virtual surgical planning improves surgical outcome measures in obstructive sleep apnea surgery. Laryngoscope. 2014;124(5):1259-66. Epub 2013/12/21. doi: 10.1002/lary.24501. PubMed PMID: 24357526.
68. Sencimen M, Bayar GR, Akcam T, Altug HA, Altug H, Gulses A, et al. Management of obstructive sleep apnea by maxillomandibular advancement surgery in an edentulous patient. The Journal of craniofacial surgery. 2012;23(6):e582-5. Epub 2012/11/23. doi: 10.1097/SCS.0b013e31826befe0. PubMed PMID: 23172485.
69. Schendel S, Powell N, Jacobson R. Maxillary, mandibular, and chin advancement: treatment planning based on airway anatomy in obstructive sleep apnea. Journal of oral and maxillofacial surgery : official journal of the American Association of Oral and Maxillofacial Surgeons. 2011;69(3):663-76. Epub 2011/03/01. doi: 10.1016/j.joms.2010.11.010. PubMed PMID: 21353928.
70. A M, T N, J T, H T, I S, H C. Good esthetic results after modified maxillomandibular advancment for obstructive sleep apnea syndrome Sleep Biol Rhythms. 2009;7(1):3-10.
71. Johnson JD, Jr., Boyd SB. Obstructive sleep apnea: a case report. The Journal of the Tennessee Dental Association. 2002;82(3):48-51. Epub 2003/02/08. PubMed PMID: 12572407.
72. El AS, El H, Palomo JM, Baur DA. A 3-dimensional airway analysis of an obstructive sleep apnea surgical correction with cone beam computed tomography. Journal of oral and maxillofacial surgery : official journal of the American Association of Oral and Maxillofacial Surgeons. 2011;69(9):2424-36. Epub 2011/05/03. doi: 10.1016/j.joms.2010.11.046. PubMed PMID: 21530047.

73. Doff MH, Jansma J, Schepers RH, Hoekema A. Maxillomandibular advancement surgery as alternative to continuous positive airway pressure in morbidly severe obstructive sleep apnea: a case report. Cranio : the journal of craniomandibular practice. 2013;31(4):246-51. Epub 2013/12/07. doi: 10.1179/crn.2013.31.4.001. PubMed PMID: 24308097.
74. Corcoran S, Mysliwiec V, Niven AS, Fallah D. Development of central sleep apnea after maxillofacial surgery for obstructive sleep apnea. Journal of clinical sleep medicine : JCSM : official publication of the American Academy of Sleep Medicine. 2009;5(2):151-3. Epub 2009/12/09. PubMed PMID: 19968049; PubMed Central PMCID: PMCPMC2670335.
75. Barrera JE, Powell NB, Riley RW. Facial skeletal surgery in the management of adult obstructive sleep apnea syndrome. Clinics in plastic surgery. 2007;34(3):565-73. Epub 2007/08/21. doi: 10.1016/j.cps.2007.04.010. PubMed PMID: 17692712.
76. W B, A C, C M, N C, Y T. Maxillomandibular advancement (MMA) for the treatment of severe obstructive sleep apnea syndrome (OSAS): The first case report in Thailand. Siriraj Med. 2007;59:369-72.
77. Conradt R, Hochban W, Brandenburg U, Heitmann J, Cassel W, Peter JH. [nCPAP therapy and maxillary and mandibular osteotomy compared: attention during the day in obstructive sleep apnea]. Wiener Medizinische Wochenschrift. 1996;146(13-14):372-4. PubMed PMID: 9012189.
78. Conradt R, Hochban W, Heitmann J, Brandenburg U, Cassel W, Penzel T, et al. Sleep fragmentation and daytime vigilance in patients with OSA treated by surgical maxillomandibular advancement compared to CPAP therapy. Journal of Sleep Research. 1998;7(3):217-23. PubMed PMID: 9785277.
79. P K, F R, H Ob, H I, Neukam FWConnell D. [Surgical management of obstructive sleep apnea]. Mund Kiefer Gesichtschir. 2007;11:81-8.
80. Zinser MJ, Zachow S, Sailer HF. Bimaxillary 'rotation advancement' procedures in patients with obstructive sleep apnea: a 3-dimensional airway analysis of morphological changes. Int J Oral Maxillofac Surg. 2013;42(5):569-78. Epub 2012/11/28. doi: 10.1016/j.ijom.2012.08.002. PubMed PMID: 23177930.
81. Jones R, J B, C J. Maxillary, mandibular and chin advancement surgery for the treatment of obstructive sleep apnea. Aus Dent J. 2010;55(3):314-21.
82. Arcuri F, Brucoli M, Benech R, Giarda M, Benech A. Maxillomandibular advancement in obstructive sleep apnea syndrome: a surgical model to investigate reverse face lift. The Journal of craniofacial surgery. 2011;22(6):2148-52. Epub 2011/11/15. doi: 10.1097/SCS.0b013e3182323dea. PubMed PMID: 22075840.
83. Van Sickels JE, Wallender A. Closure of anterior open bites with mandibular surgery: advantages and disadvantages of this approach. Oral and maxillofacial surgery. 2012;16(4):361-7. Epub 2012/09/05. doi: 10.1007/s10006-012-0361-6. PubMed PMID: 22945345.
84. Conley RS, Legan HL. Correction of severe obstructive sleep apnea with bimaxillary transverse distraction osteogenesis and maxillomandibular advancement. American journal of orthodontics and dentofacial orthopedics : official publication of the American Association of Orthodontists, its constituent societies, and the American Board of Orthodontics. 2006;129(2):283-92. Epub 2006/02/14. doi: 10.1016/j.ajodo.2005.11.029. PubMed PMID: 16473723.
85. WB C. Comprehensive reconstructive surgery for obstructive sleep apnea. J Ky Med Assoc. 2004;102(4):154-62.
